# Demographic and Methodological Heterogeneity in Electrocardiogram Signals From Guinea Pigs

**DOI:** 10.3389/fphys.2022.925042

**Published:** 2022-06-02

**Authors:** Kazi T. Haq, Blake L. Cooper, Fiona Berk, Anysja Roberts, Luther M. Swift, Nikki Gillum Posnack

**Affiliations:** ^1^ Sheikh Zayed Institute for Pediatric Surgical Innovation, Washington D.C., DC, United States; ^2^ Children’s National Heart Institute, Washington D.C., DC, United States; ^3^ Department of Pharmacology & Physiology, Washington D.C., DC, United States; ^4^ Department of Pediatrics, Washington D.C., DC, United States

**Keywords:** electrocardiogram (ECG), cardiac electrophysiology, vectorcardiogram (VCG), age-dependent, sex-dependent

## Abstract

Electrocardiograms (ECG) are universally used to measure the electrical activity of the heart; however, variations in recording techniques and/or subject demographics can affect ECG interpretation. In this study, we investigated variables that are likely to influence ECG metric measurements in cardiovascular research, including recording technique, use of anesthesia, and animal model characteristics. Awake limb lead ECG recordings were collected *in vivo* from adult guinea pigs using a platform ECG system, while recordings in anesthetized animals were performed using both a platform and needle ECG system. We report significant heterogeneities in ECG metric values that are attributed to methodological differences (e.g., ECG lead configuration, ECG recording platform, presence or absence of anesthesia) that persist even within the same cohort of animals. Further, we report that variability in animal demographics is preserved in *vivo* ECG recordings—with animal age serving as a significant contributor, while sex-specific influences were less pronounced. Methodological approaches and subject demographics should be fully considered when interpreting ECG values in animal models, comparing datasets between studies, or developing artificial intelligence algorithms that utilize an ECG database.

## Introduction

Demographic heterogeneity in human body surface electrocardiogram (ECG) signals has been well documented (Sia et al., 2019; Mirahmadizadeh et al., 2021). Factors such as age (Bachman et al., 1981), sex (Simonson et al., 1960), or race (Rautaharju et al., 1994) have been shown to modulate the morphology of an ECG signal, which can impact ECG parameter measurements and data interpretation in both clinical practice and cardiovascular research. However, animal studies that focus on *in vivo* assessment of ECG recordings often have insufficient demographic details (Quinn et al., 2011). This lack of information can affect the candidacy of an animal model to predict human cardiovascular behavior as it relates to normal cardiac physiology, pathophysiology, or drug safety and efficacy testing. Guinea pigs have been used as a translational model for preclinical assessment of cardiac electrophysiology (Greer-Short et al., 2017; Cooper et al., 2021; Wu et al., 2021) and arrhythmia susceptibility (Woulfe et al., 2018), due in part to similarities in ionic currents, cardiac action potential shape, and the influence of excitation-contraction coupling (Luo and Rudy, 1994; Bers, 2000; Edwards and Louch, 2017). Indeed, a validation study reported that guinea pig models have a high level of specificity and sensitivity in predicting clinical outcomes to reference compounds known to widen the QRS complex, lengthen QT duration, or precipitate heart block (Guo et al., 2009). Yet, the utility of experimental models and/or data comparison between studies can be hindered by a shortage of information on age- and sex-specific differences in guinea pig models.

Similarly, different technical approaches to recording *in vivo* ECG signals can introduce heterogeneity in parameter measurements. The majority of human ECG recordings are carried out in a clinical setting, using a standard 12-lead ECG system. However, no such standard procedure exists for recording *in vivo* ECG signals from experimental animals. Indeed, ECG platforms and technical approaches can vary according to the species of choice (e.g., platform ECG device (Ho et al., 2011), telemetric ECG (Rossi et al., 2014), or needle electrode (Brisinda et al., 2007) ECG). Within the current literature, heart rate measurements in age-matched male guinea pigs can vary by ∼17% across different technical approaches (Brisinda et al., 2007; Brouillette et al., 2007; Hess et al., 2007; Botelho et al., 2016). The lack of studies focused on the heterogeneity of recording methodologies can contribute to the ‘reproducibility crisis’, influence ECG recording interpretation, and limit the broad utility of experimental data sets (Ramirez et al., 2017).

In the current study, we collected and analyzed cardiac ECGs that were acquired from adult guinea pigs using three different experimental approaches: platform ECG system with awake animals (PES-AW), a platform ECG system with anesthetized animals (PES-AN) and a needle electrode system with anesthetized animals (NES-AN). Using the latter, we also addressed whether ECG lead configuration and/or construction of frontal plane vectorcardiogram (VCG) impacts measured ECG values and data interpretation. Finally, we explored the impact of model selection on ECG signal morphology and data analysis by comparing age- and sex-specific differences using the guinea pig model. Collectively, the results of our study indicate that data collection methods and animal characteristics alter ECG parameters (e.g., QT interval, QRS duration, PR interval) that are commonly employed for drug safety testing, assessment of cardiac physiology and pathophysiology, or the development of artificial intelligence (AI) for ECG analysis. To improve the rigor and reproducibility of cardiac electrophysiology studies, care should be taken to provide sufficient detail on the experimental approaches and study subjects so that datasets can be compared between studies.

## Materials and Methods

### Animal Model

Animal procedures were approved by the Institutional Animal Care and Use Committee of the Children’s Research Institute and followed the National Institutes of Health’s Guide for the Care and Use of Laboratory Animals. Electrocardiograms were recorded from Dunkin Hartley guinea pigs (Hilltop Lab Animals, Scottsdale PA, n = 30), as described below. To investigate age-dependent effects on cardiac electrophysiology, animals were separated into a younger adult group (51 ± 6 days old, 380 ± 69 g body weight, n = 16) and older adult group (620 ± 2 days old, 897 ± 114 g body weight, n = 14). Additional studies were performed to examine ECG heterogeneity due to sex-specific differences (n = 14 females, n = 16 males). At the end of study, all animals were euthanized by exsanguination under anesthesia.

### ECG Recording Techniques

**FIGURE 1:**
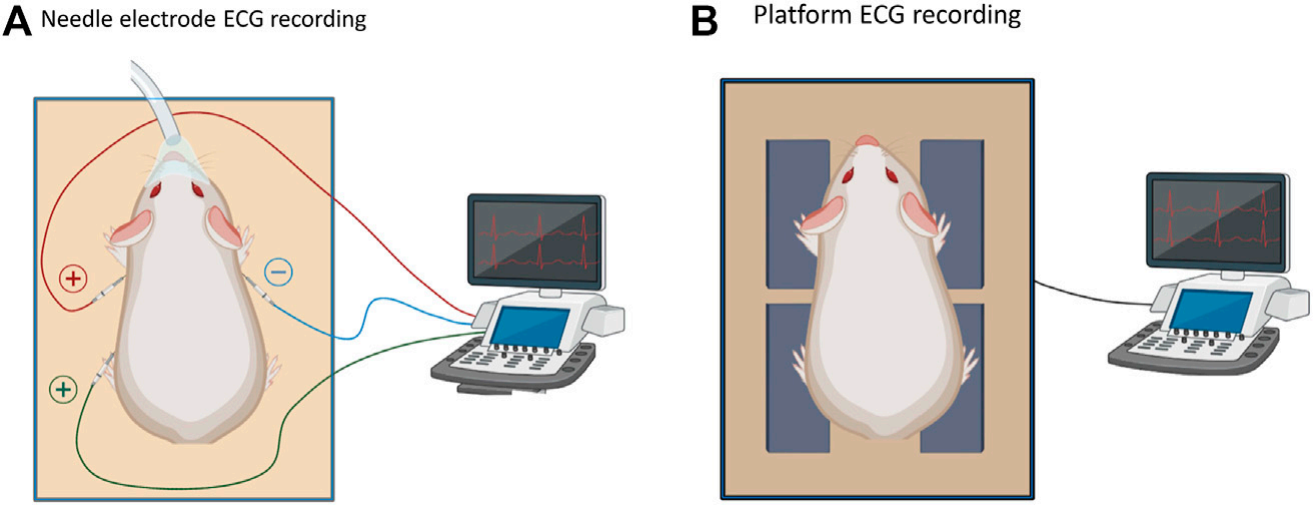
Methodological effect on ECG parameters. **(A)**. Schematic of a needle ECG system to record ECGs in an anesthetized state. **(B)**. Platform ECG system to record ECG signals in either an awake or anesthetized state.

ECG signals were recorded from both awake and isoflurane-anesthetized animals. Awake recordings were collected using a platform ECG system (ecgTUNNEL: emka Technologies, Sterling VA), while recordings in anesthetized animals were performed using both the platform ECG system and a needle electrode system to record limb lead ECG signals (Figure 1). The platform ECG system is comprised of electrode pads for the placement of an animal’s paws in a supine position (Romanowicz et al., 2021). Ultrasound gel was applied to the surface of the electrode pads and a barrier was placed underneath the animal’s midsection to reduce noise. Under awake conditions, animals were allowed to acclimate for 5–10 min before recording a ∼1 min ECG using a lead I configuration (left front pad—positive, right front pad—negative electrode). A low pass filter (59 Hz, analog; emka Technologies) was applied, and ECG signals were recorded using ecgAUTO software (emka Technologies). Animals were then anesthetized with 3–4% isoflurane for 10 min, and ECG signals were collected again using the platform ECG system while maintaining anesthesia.

For needle ECG recordings, animals were anesthetized with 3–4% isoflurane for 10 min, and needle electrodes were placed subcutaneously as shown in to collect ECG signals (lead I, II) (Hennis et al., 2022). Biosignals were collected using a PowerLab acquisition system and LabChart 8 software (ADInstruments, Colorado Springs CO). A high pass filter (10 Hz, analog), low pass filter (100 Hz, analog), gain amplifier (1k) were applied using a differential amplifier (DP-304A Warner, Holliston MA) in conjunction with a 60 Hz noise eliminator (Hum Bug: Digitimer, Fort Lauderdale FL). Care was taken to acquire ECGs after the same length of time and depth of isoflurane anesthesia, which is a fast-acting and short-lasting anesthetic agent (Lindsey et al., 2018).

### Platform ECG System: Data Analysis

ECG signals (lead I) collected from awake (PES-AW) and anesthetized (PES-AN) animals were analyzed in ecgAUTO 5.5.6 (emka Technologies). Parameters of interest included RR interval, heart rate, *p* duration, PR interval, QRS duration, QT interval and QTc (Bazett corrected (Bazett, 1997)). From each ECG recording, 15 beats were analyzed and averaged (5 consecutive beats from three different time intervals from ∼1 min recording). Each beat was reviewed for correct fiducial markers by two independent reviewers (KH and FB) to prevent reader bias (Panicker et al., 2009). A tangent rule was applied in instances where the end of a fiducial point did not clearly intersect the isoelectric line (Hermans et al., 2017; Rosenthal et al., 2018).

### Needle ECG System: Data Analysis

ECG signals (lead I, II) collected from isoflurane anesthetized animals (NES-AN) were acquired in Labchart 8.0 (ADInstruments). Biosignals were imported into MATLAB R2021a (MathWorks Inc, Natick MA) for baseline drift removal using a continuous wavelet transform function, and frontal plane leads (lead III, aVL, aVR, aVF) were calculated from standard limb leads I and II using Einthoven’s law (for review (Kligfield et al., 2007)). 6-lead data was imported into Labchart 8.0 as a text file, wherein a representative waveform library was constructed and applied to 10 consecutive beats. ECG fiducial points were verified by two independent reviewers (KH, BC).

### VCG Analysis

A custom MATLAB script was used to construct frontal plane VCGs, as previously described (Haq et al., 2021a; Haq et al., 2021b; Haq et al., 2021c), with lead I as the x-axis and aVF as the *y*-axis. First, 10 consecutive beats were aligned at their corresponding R-peaks to generate an average waveform. Fiducial point markers (Q-onset, R-peak, T-peak, and T-end) were independently verified and corrected (KH). Then, six VCG parameters were calculated: spatial ventricular gradient (SVG) magnitude, SVG elevation, QRS-elevation, T-elevation, Wilson ventricular gradient (WVG) and QRST angle.

### Statistical Analysis

Statistical analysis was performed using either a homoscedastic Student’s t-test (two groups) or analysis of variance (three or more groups). Significance was defined by a *p*-value <0.05, or an adjusted *p*-value after correction for multiple comparisons using a two-stage linear step-up procedure to control the false discovery rate using a 0.05 cutoff (Benjamini et al., 2006). Results are reported as mean ± SD. Significance is denoted in figures with an asterisk (*p < 0.05, **p < 0.01, ***p < 0.001, ****p < 0.0001).

## Results

### Establishing an Average ECG Beat

Before performing subsequent analysis, we examined ECG recordings to determine the appropriate number of beats needed to construct an average ECG beat. Using the platform ECG system, an average ECG beat was similar when generated from either 10 or 20 consecutive beats (Supplementary Figure S1). However, intra-recording variability was observed between three different time points (beginning, middle, end) within a ∼1 min recording using the platform system. To exclude this variability bias, all platform ECG recordings were analyzed by averaging five consecutive beats across the aforementioned three time points, as previously described (Botelho et al., 2016). In comparison, needle ECG recordings showed negligible variability across the three different time points (data not shown). Accordingly, data analysis of needle-based ECG signals was performed using 10 consecutive beats to construct an average ECG beat for further analysis.

### Recording Methodology Contributes to ECG Signal Heterogeneity

**FIGURE 2:**
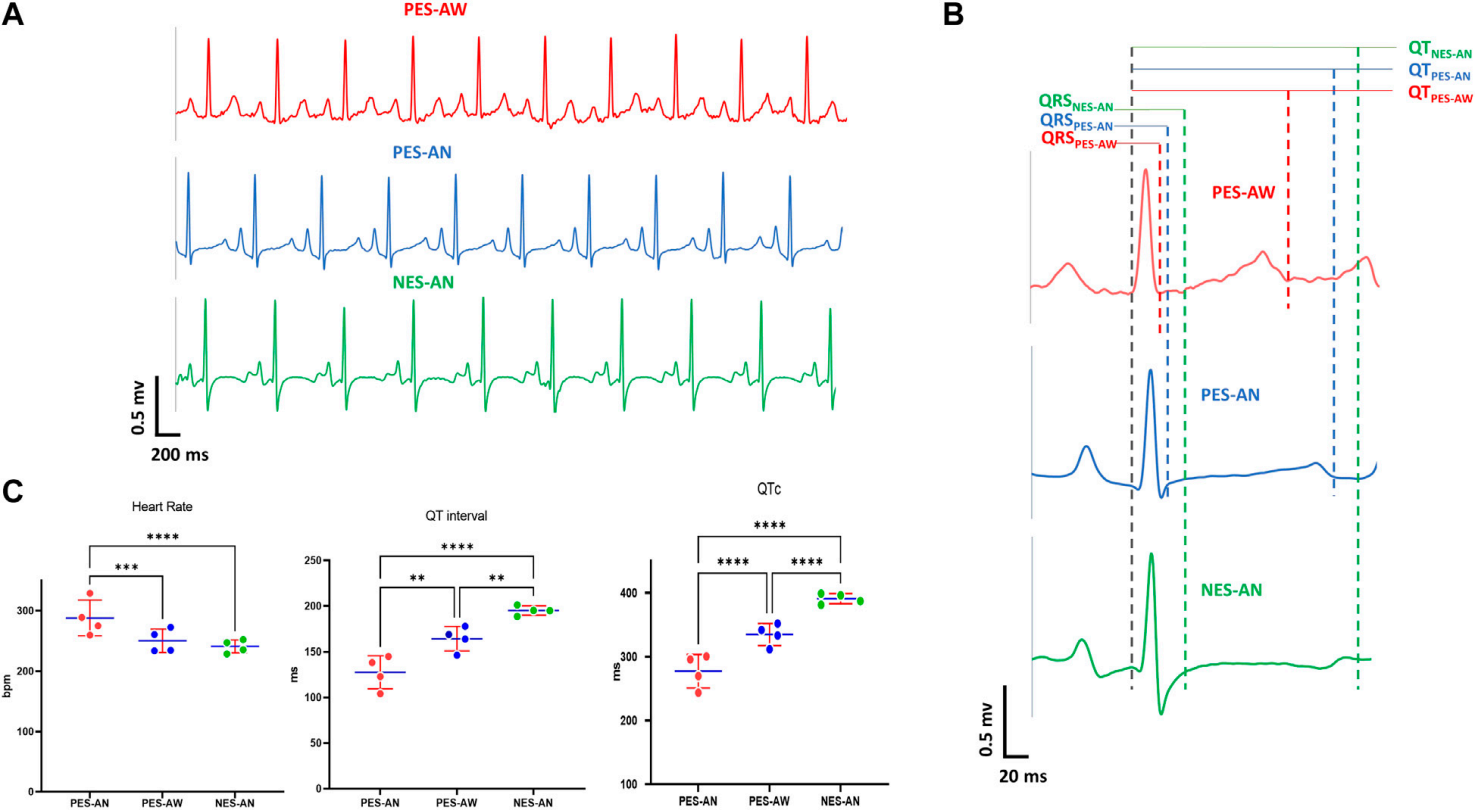
Effect of recording methodology on ECG parameters. **(A)**. Example lead I ECG traces of a young guinea pig acquired by three methods: PES-AW, PES-AN, and NES-AN. **(B)**. A representative ECG beat of a young female guinea pig from PES-AW, PES-AN, and NES-AN. **(C).** Comparison of three ECG metrics (heart rate, QT interval, and QTc) from PES-AW, PES-AN, and NES-AN. 2-way ANOVA with 0.05 FDR cutoff was used to calculate the *p* values. ∗∗*p* < 0.01, ∗∗∗*p* < 0.001, ∗∗∗∗*p* < 0.0001.

We tested whether ECG signals were preserved across methodologies (PES-AW, PES-AN, NES-AN) using the same animal cohort. As an example, ECG traces (lead I) from a female guinea pig show significant morphological differences between ECG signals collected using these three different methodologies (Figure 2). Of note, the S-wave was nearly abolished in the PES-AW, but it became progressively more prominent in the PES-AN and NES-AN traces. Further, the ST segment was elevated in the PES-AN trace, but not the PES-AW or NES-AN traces. Finally, the T-wave was biphasic in the NES-AN recordings, but monophasic and positively deflected in both the PES-AW and PES-AN signals. A qualitative comparison between three average beats are shown (Figure 2B), which demonstrate differences in QRS and QT measurements (QRS_NES-AN_ > QRS_PES-AN_ > QRS_PES-AW;_ QT_NES-AN_ > QT_PES-AN_ > QT_PES-AW_).

For a quantitative comparison, ECG signals from age-matched, young adult female guinea pigs (*n* = 4) were compared (Figure 2C). Within this cohort, the three different methodological approaches yielded statistically significant differences in heart rate, QT, and QTc parameters. As expected, heart rate was faster in awake versus anesthetized conditions (PES-AW: 288.3±25.6 bpm, PES-AN: 250.5±16.8 bpm, *p* < 0.001; PES-AW: 288.3±25.6 bpm, NES-AN: 241.1±9.3 bpm, *p* < 0.001). QT interval and QTc were significantly longer in NES-AN, compared with PES-AN and PES-AW (NES-AN: 195.1±4.4 ms; PES-AN: 164.3±11.6 ms, *p* < 0.001, NES-AN: 195.1±4.4 ms; PES-AW: 127.6±15.7 ms, *p* < 0.0001). Similarly, QTc was also found to be significantly different among the three recording methods, with a longer QTc observed in NES-AN (Figure 2C and Table 1).

**TABLE 1:** Effect of three different recording methods on ECG parameters in young female guinea pigs.

Group	RR(ms)	HR(BPM)	PR(ms)	Pdur(ms)	QRS(ms)	QT(ms)	QTc (ms)
PES-AW	210.4±18.3	288.3±25.6	58±4.4	39.3±5.4	41±8.1	127.6±15.7	277.4±22.8
PES-AN	240.7±16.1	250.5±16.8	49.8±3.3	34±4.5	37.7±3.7	164.3±11.6	334.8±14.8
NES-AN	249.3±9.7	241.1±9.3	53.4±7.3	22.1±4	51.1±12.9	195.1±4.4	390.9±7

Values are means ± SD in ms or bpm (n = 4 animals).

We also observed significant age-dependent differences in ECG parameters between the three methodological approaches (Table 2). In the older adult group, NES-AN signals had a longer PR interval (NES-AN: 62.9±6.6 ms, PES-AN: 54.3±3 ms, *p* < 0.05), QT (NES-AN: 211.5±19 ms, PES-AN: 193.9±8 ms, *p* < 0.05) and QTc (NES-AN: 383.7±19.6 ms, PES-AN: 359.8±4.9 ms, *p* < 0.05), but a shorter P duration (PES-AN: 40.6±2.4 ms, NES-AN: 24.1±2.3, *p* < 0.0001) when compared with PES-AN signals. In the younger adult group, heart rate (PES-AN: 261.9±23 bpm , NES-AN: 247.4±16.9 bpm, *p* < 0.05) and P duration (PES-AN: 34.7±4.7 ms, NES-AN: 22.7±2.9 ms, *p* < 0.0001) were shorter in NES-AN compared to PES-AN, while PR interval (NES-AN: 56.1.6±7.2 ms, PES-AN: 48.7±4.5 ms, *p* < 0.05), QRS duration (NES-AN: 63.5±12.8 ms, PES-AN: 44±7.8 ms, *p* < 0.0001), QT interval (NES-AN: 179.7±15 ms, PES-AN: 162.6±14.4 ms, *p* < 0.05), and QTc (NES-AN: 363.9±22.3 ms, PES-AN: 337.9±19 ms, *p* < 0.05) were longer in NES-AN versus PES-AN. Furthermore, nearly all of the ECG parameters showed significant differences between NES-AN and PES-AW in both older and younger adult groups (Table 2).

**TABLE 2:** Methodological effect on ECG parameters in younger and older adult guinea pigs.

Group	RR(ms)	HR(BPM)	PR(ms)	Pdur(ms)	QRS(ms)	QT(ms)	QTc(ms)
old NES-AN(*n* = 9)	304.3±31.9	200.7±24.6	62.9±6.6	24.1±2.3	77.8±12.4	211.5±19	383.7±19.6
old PES-AN (*n* = 9)	291.1±20.5	207.6±14.8	54.3±3	40.6±2.4	71±14	193.9±8	359.8±4.9
*p*-value	0.2	0.2	**<0.05**	**<0.0001**	0.05	**<0.05**	**<0.05**
young NES-AN (*n* = 13)	243.6±16.6	247.4±16.9	56.1±7.2	22.7±2.9	63.5±12.8	179.7±15	363.9±22.3
young PES-AN (*n* = 13)	231.5±21	261.9±23	48.7±4.5	34.7±4.7	44±7.8	162.6±14.4	337.9±19
*p*-value	0.065	**<0.05**	**<0.05**	**<0.0001**	**<0.0001**	**<0.05**	**<0.05**
old NES-AN (*n* = 6)	315.1±37.6	193±22.6	60±3.3	23.3±2.7	83.1±11.6	216.5±19.8	386±22.5
old PES-AW (*n* = 6)	244.4±32	250±31.9	61±5.5	38.4±3.7	60.7±6	150.9±13.6	305.7±11.9
*p*-value	**<0.001**	**<0.05**	0.36	**<0.001**	**<0.05**	**<0.001**	**<0.05**
young NES-AN (*n* = 7)	241.2±15.3	249.8±16.8	55.4±6.2	22.5±3.2	60.3±15.8	181±18.1	368.1±28.1
young PES-AW (*n* = 7)	215.5±21.7	282.1±28.3	58.9±4.2	38.8±4.9	45.3±8.4	132.9±14	286.1±20.1
*p*-value	**<0.05**	**<0.05**	0.16	**<0.0001**	0.06	**<0.001**	**<0.0001**

Values are means ± SD in ms or bpm. *p* value is calculated by homoscedastic Student’s t-test.

*p* values that are statistically significant

### Anesthesia Contributes to ECG Signal Heterogeneity

**FIGURE 3:**
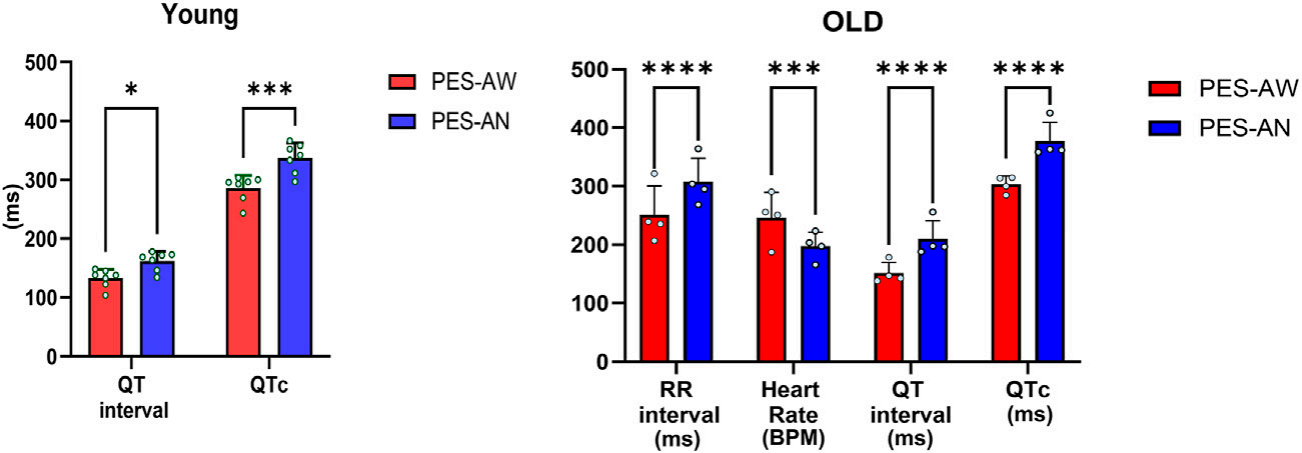
Effect of anesthesia on ECG parameters. Comparisons were made both in younger (n = 7) and older (n = 4) adult animals in awake (PES-AW) and anesthetized condition (PES-AN). 2-way ANOVA with 0.05 FDR cutoff was used to calculate the *p* values. ∗*p* < 0.05, ∗∗∗*p* < 0.001, ∗∗∗∗*p* < 0.0001.

Using the platform ECG recording approach, we compared ECG signals from both awake and isoflurane-anesthetized animals to investigate the impact of anesthesia on age-matched animals (Hauser et al., 2005; Yang et al., 2014; Konopelski and Ufnal, 2016; Schmitz et al., 2016). In the young adult group, the QT interval (PES-AN: 162.4±14.8 ms, PES-AW: 132.9±14 ms, *p* < 0.05) and QTc (PES-AN: 337.3±23.3 ms, PES-AW: 286.1±20.1 ms, *p* < 0.001) were significantly longer in anesthetized animals compared to the awake animals (Table 3). As shown in Figure 3, similar differences were also observed in the older adult group, wherein anesthetized animals had a longer QT (PES-AN: 209.6±27.1 ms, PES-AW: 151.6±15.9 ms, *p* < 0.05) and QTc (PES-AN: 377.2±27.8 ms, PES-AW: 303.5±12.3 ms, *p* < 0.05), and a slower heart rate compared with awake animals (PES-AN: 197.5±20.8 bpm, PES-AW: 246.1±37.4 bpm, *p* < 0.05).

**TABLE 3:** Effect of anesthesia on ECG parameters in younger and older adult guinea pigs, recorded using a platform ECG system.

Group	RR(ms)	HR(BPM)	PR(ms)	Pdur(ms)	QRS(ms)	QT(ms)	QTc(ms)
Young PES-AW(*n* = 7)	215.5±21.7	282.1±28.3	58.9±4.2	38.8±4.9	45.3±8.4	132.9±14	286.1±20.1
Young PES-AN (*n* = 7)	231.5±17.9	260.8±19.9	49.4±2.8	33.9±4.1	44.1±8.6	162.4±14.8	337.3±23.3
*p* value	0.09	0.08	**<0.001**	**<0.05**	0.4	**<0.05**	**<0.001**
Old PES-AW (*n* = 4)	251±42.8	246.1±37.4	59.8±5.4	39.5±4.4	62.1±3.9	151.6±15.9	303.5±12.3
Old PES-AN (*n* = 4)	308±34.9	197.5±20.8	55.2±3.2	41.4±1.7	66.4±13.5	209.6±27.1	377.2±27.8
*p* value	0.06	**<0.05**	0.12	0.25	0.31	**<0.05**	**<0.05**

Values are means ± SD in ms or bpm. *p* value is calculated by homoscedastic Student’s t-test.

*p* values that are statistically significant

### ECG Measurements Vary by Lead Configuration

**FIGURE 4:**
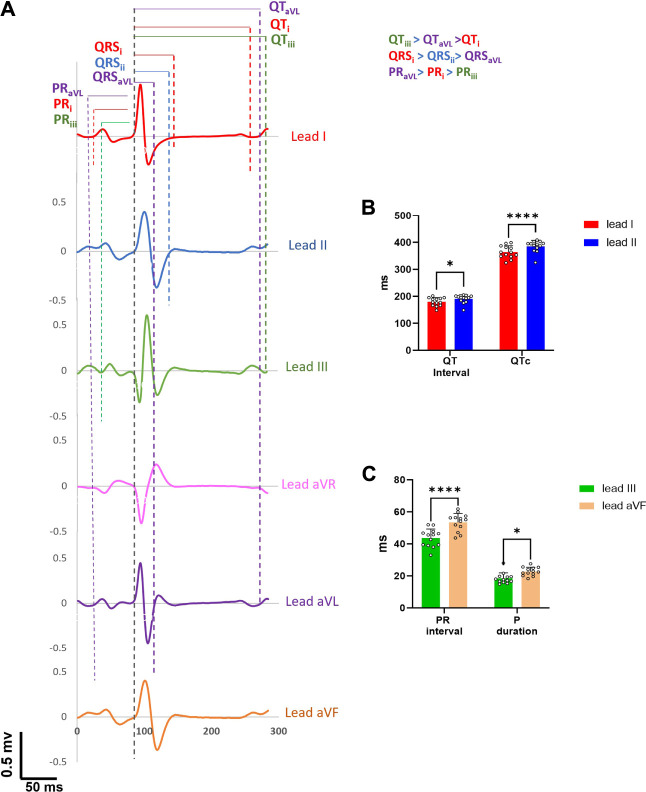
Inter-beat variability on ECG parameters. **(A)**. One representative beat from six frontal leads of a young guinea pig show marked differences in ECG parameter values. **(B)**. QT intervals and QTc intervals in lead II are significantly longer than in lead I (n = 13). **(C).** PR interval and *p* duration measured in lead aVF are significantly longer than lead III. 2-way ANOVA with 0.05 FDR cutoff was used to calculate the *p* values. ∗*p* < 0.05, ∗∗∗∗*p* < 0.0001.

Using the needle ECG recording approach, we next compared whether measured ECG parameters varied by lead configuration using six ECG leads in the frontal plane. Most of the ECG parameters (PR interval, P duration, QRS interval, QT interval and QTc) showed significant differences when measured from different leads, both in the older and younger adult groups **(** Supplementary Table S1-S2). For example, P duration (lead I: 22.7±2.9 ms; lead II: 26.7±2.9 ms; *p* < 0.05), QT interval (lead I: 179.7±15 ms, lead II: 190.3±14.8 ms, *p* < 0.05), and QTc (lead I: 363.9±22.3 ms, lead II: 385.4±21.1 ms, *p* < 0.05) were significantly longer in lead II compared to lead I, while QRS duration (lead I: 63.5±12.8 ms, lead II: 56.6±5 ms, *p* < 0.05) was shorter in younger animals. An example of individual single beats (same time window) collected from a young male guinea pig is shown in Figure 4, wherein significant deviations in ECG measurements are displayed between lead configuration (QT_iii_ > QT_aVL_ > QT_i;_ QRS_i_ > QRS_ii_ > QRS_aVL ;_PR_aVL_ > PR_i_ > PR_iii_ ). Figures 4B,C further illustrate ECG signal variability depending on the lead measurement (lead I vs lead II: QT_ii_>QT_i_; QTc_ii_>QTc_i_; lead III vs lead aVF: PR interval aVF > PRinterval_iii_; P-duration aVF > P-duration_iii_).

### Age-dependent Effects on ECG Measurements

**FIGURE 5:**
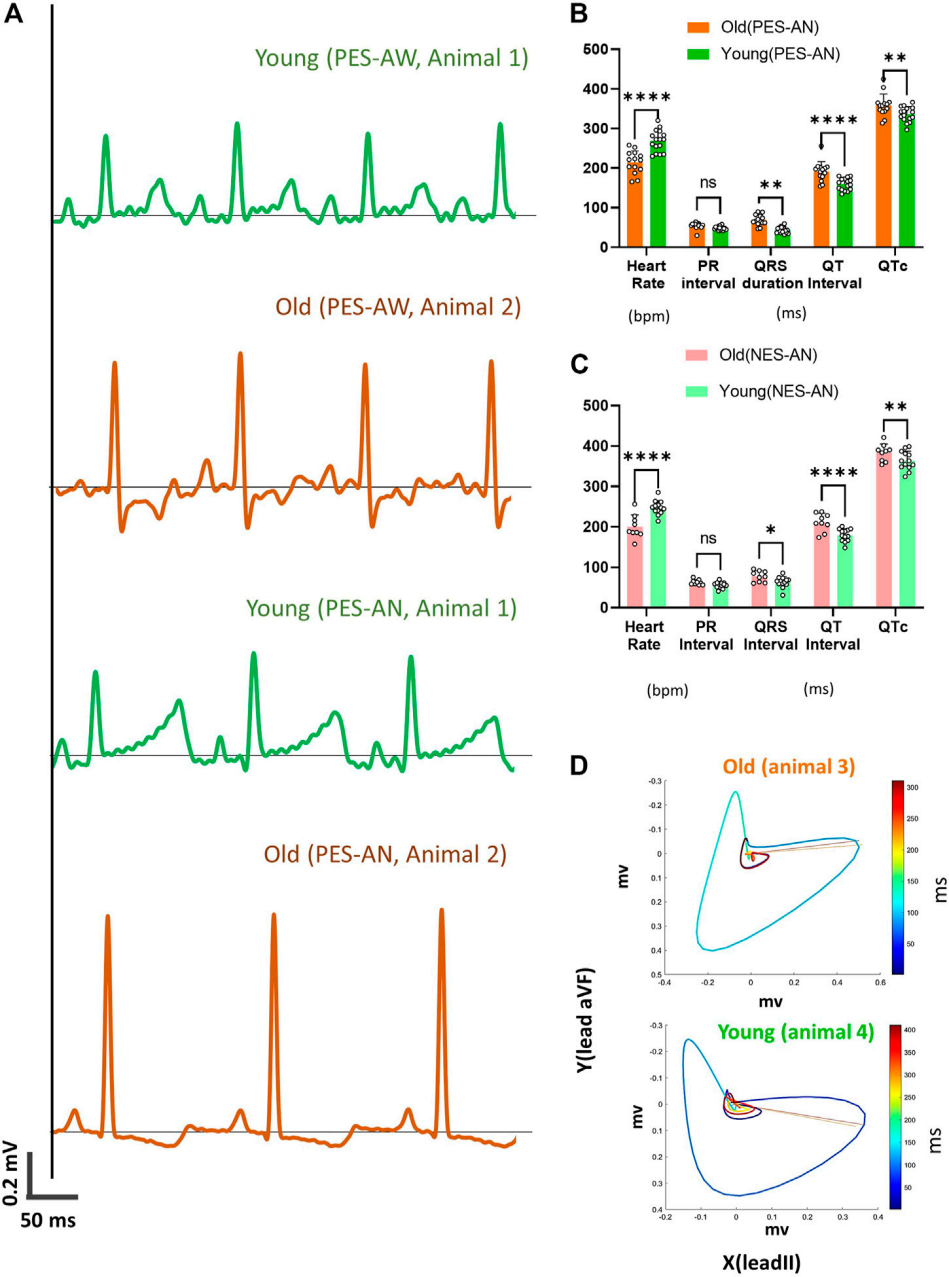
Age-dependent effects on ECG parameters. **(A)**. Example lead I ECG traces acquired from a young female and old female guinea pig using a platform ECG system. **(B)**. Comparison of different ECG metrics from old (*n* = 14) and young (*n* = 16) animals using PES-AN. **(C)**. Comparison of different ECG metrics from old (*n* = 9) and young (*n* = 13) animals using NES-AN. 2-way ANOVA with 0.05 FDR cutoff was used to calculate the *p* values. **(D)**. Frontal plane VCG in a younger and older adult animal. ∗*p* < 0.05, ∗∗*p* < 0.01, ∗∗∗*p* < 0.001, ∗∗∗∗*p* < 0.0001.

To investigate the impact of model selection and age-dependent effects on ECG measurements, guinea pigs were grouped by age (younger adult and older adult). Representative ECG traces are shown (Figure 5), illustrating significant morphological differences between a young adult and older adult guinea pig, under both awake and anesthetized conditions using the platform ECG approach. Under awake conditions (PES-AW), older animals exhibited more prominent S waves and thus, a wider QRS duration compared to younger adults—Moreover, the T-wave was bi-phasic in older adult animals, but only a positively deflected T-wave was recorded in the younger adult animals. When under anesthesia (PES-AN), the QRS amplitude remained larger in the older adult animals, but unlike the awake conditions, the S wave was almost completely abolished. Prominent ST elevation was observed in the T-wave of the younger animals. Similar to awake conditions, the younger animals displayed a positively deflected T-wave, while older animals maintained a bi-phasic T-wave—albeit the negative deflection became more prominent.

In agreement to the ECG morphological dissimilarities, younger and older adult guinea pigs had significant differences in measured ECG parameters using the PES-AN approach (Figure 5B; Table 4). Heart rate was slower in older versus younger adults (Old: 214.9±27.6 bpm; Young: 269.1±26.4 bpm; *p* < 0.0001). Compared with younger adults, older animals had prolonged a PR interval (Old: 55.1±7.9; Young: 49±4.8 ms; *p* < 0.05), QRS duration (Old: 68.5±12.8 ms; Young: 43.7±7.8 ms; *p* < 0.0001), QT interval (Old: 191.3±24.1 ms; Young: 159.6±14.8 ms; *p* < 0.0001), and QTc (Old: 358.6±27.7 ms; Young: 336.1±18.3 ms; *p* < 0.05). Similar trends were observed in the PES-AW recorded ECGs (Table 5).

**TABLE 4:** Age-dependent effects on ECG parameters using PES-AN approach.

Group	RR(ms)	HR(BPM)	PR(ms)	Pdur(ms)	QRS(ms)	QT(ms)	QTc(ms)
Old(n = 14)	284.6±39.3	214.9±27.6	55.1±7.9	38.1±5.8	68.5±12.8	191.3±24.1	358.6±27.7
Young(n = 16)	225.7±22.9	269.1±26.4	49±4.8	35.8±5	43.7±7.8	159.6±14.8	336.1±18.3
*p* value	**<0.0001**	**<0.0001**	**<0.05**	0.13	**<0.0001**	**<0.0001**	**<0.05**

Values are means ± SD in ms or bpm. *p* value is calculated by homoscedastic Student’s t-test.

*p* values that are statistically significant

**TABLE 5:** Age-dependent effects on ECG parameters using PES-AW approach.

Group	RR(ms)	HR(BPM)	PR(ms)	Pdur(ms)	QRS(ms)	QT(ms)	QTc(ms)
Old(*n* = 7)	255.5±40.2	241±36.8	61.1±5.1	39.6±4.5	61.7±6.2	154.8±15.9	307±14.6
Young(*n* = 7)	215.5±21.7	282.1±28.3	58.9±4.2	38.8±4.9	45.3±8.4	132.9±14	286.1±20.1
*p* value	**<0.05**	**<0.05**	0.22	0.39	**<0.05**	**<0.05**	**<0.05**

Values are means ± SD in ms or bpm. *p* value is calculated by homoscedastic Student’s t-test.

*p* values that are statistically significant

Using the NES-AN approach, age-dependent effects were also measured from the six frontal leads (Table 6). When compared with younger adults, lead I recordings from the older adults showed significant prolongation of the PR interval (Old: 62.9.±6.6 ms; Young: 56.1±7.2 ms; *p* < 0.05), QRS duration (Old: 77.8±12.4; Young: 63.5±12.8 ms; *p* < 0.0001), QT interval (Old: 211.5±19 ms; Young: 179.7±15 ms; *p* < 0.05), and QTc (Old: 383.7±19.6 ms; Young: 363.9±22.3 ms; *p* < 0.05). The constructed frontal plane VCG parameters did not show any statistically significant age-dependent effects (Table 7), however, as shown in Figure 5D, the QRS vector tended to lift upward in the older animals (Q_elevation: Old: −9.53±13.9 deg; Young: −6.23±12.49 deg; *p* = 0.31) and there was a decrease in the QRST angle (QRST angle: Old: 56.86±64.22 deg; Young: 66.01±49.62 deg; *p* = 0.37).

**TABLE 6:** Age-dependent effects on ECG parameters from different leads, using NES-AN approach.

Lead	RR(ms)	HR(BPM)	PR(ms)	Pdur(ms)	QRS(ms)	QT(ms)	QTc(ms)
I (old, *n* = 9)	304.3±31.9	200.7±24.6	62.9±6.6	24.1±2.3	77.8±12.4	211.5±19	383.7±19.6
I (young, *n* = 13)	243.6±16.6	247.4±16.9	56.1±7.2	22.7±2.9	63.5±12.8	179.7±15	363.9±22.3
*p* value	**<0.0001**	**<0.001**	**<0.05**	0.21	**<0.05**	**<0.05**	**<0.05**
II(old)			65.2±3.1	31.8±3.8	59.1±3.3	214.3±13.5	390.1±9
II(young)			55.9±9.5	26.7±2.9	56.6±5	190.3±14.8	385.4±21.1
*p* value			**<0.05**	**<0.05**	0.15	**<0.05**	0.32
III(old)			55.2±6.7	22.6±4.3	54.3±3.7	208.7±14.6	379.9±14.9
III(young)			43.7±5.4	18.5±3.3	53.2±5.2	187.8±15.7	380.3±23.2
*p* value			**<0.001**	**<0.05**	0.34	**<0.05**	0.49
aVR(old)			66.5±3.7	30.3±3.9	63.9±2.5	214.3±14	390±10.3
aVR(young)			55±8.2	25.3±6.8	61.1±5.8	180.8±19.9	365.7±30.9
*p* value			**<0.05**	0.07	0.15	**<0.05**	**<0.05**
aVL(old)			54.9±6.7	18.9±2.6	46.5±10.1	204.7±14	372.7±16.5
aVL(young)			54.6±6.4	17.5±2.5	39.9±10.9	177.8±19.9	359.6±30.3
*p* value			0.47	0.15	0.12	**<0.05**	0.18
aVF(old)			60.5±7.3	28.5±1.6	57.5±5.3	211.9±13.6	385.6±10
aVF(young)			53.5±5.3	22.8±2.6	51.7±8.1	185.1±14.5	374.8±19.2
*p* value			**<0.05**	**<0.0001**	0.07	**<0.05**	0.12

Values are means ± SD in ms or bpm. *p* value is calculated by homoscedastic Student’s t-test.

*p* values that are statistically significant

**TABLE 7:** Age-dependent effects on VCG parameters using NES-AN approach.

Groups	SVG_Mag (Mv)	SVG_elevation (deg)	Q_elevation (deg)	T_elevation (deg)	WVG (Mv-Ms)	QRSTang (deg)
old(*n* = 6)	0.53±0.13	−10.77±13.87	−9.53±13.9	−1.28±23.97	0.64±0.35	56.86±64.22
Young(*n* = 13)	0.55±0.26	−6.89±11.62	−6.23±12.49	−25.32±43.06	0.55±0.53	66.01±49.62
*p* value	0.46	0.27	0.31	0.12	0.370	0.37

Values are means ± SD. *p* value is calculated by homoscedastic Student’s t-test.

### Sex-dependent Effects on ECG Parameters

**FIGURE 6:**
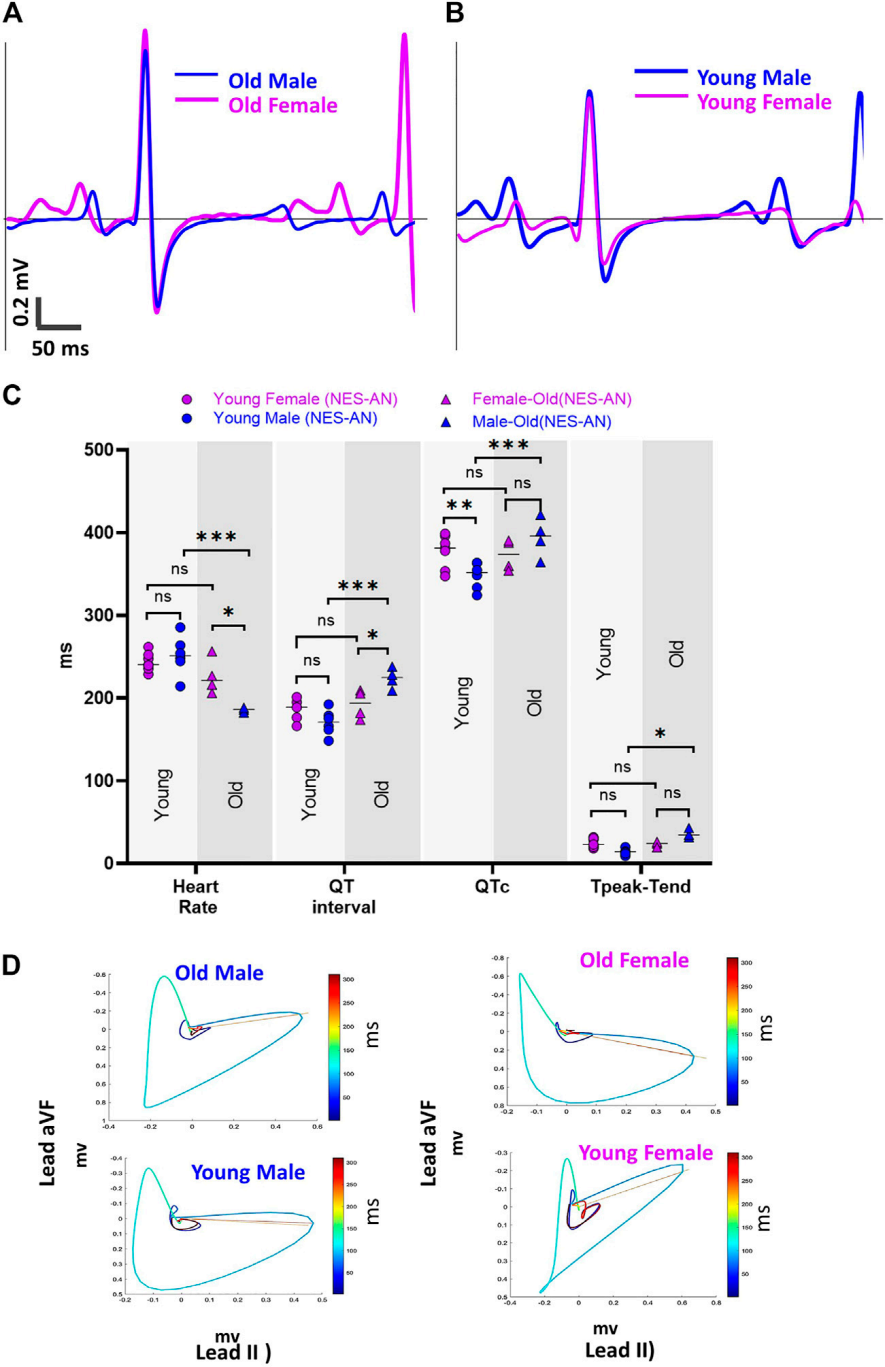
Sex effect on ECG parameters. **(A)** Example lead I ECG traces from an old male and old female guinea pig in NES-AN. **(B)** Example lead I ECG traces from a young male and young female guinea pig in NES-AN. **(C).** Comparison of ECG parameters in males (*n* = 4) and females (*n* = 4) in old and young age groups. **(D).** Example VCG loops from a male and female animal from old and young age groups. 2-way ANOVA with 0.05 FDR cutoff was used to calculate the *p* values. ∗*p* < 0.05, ∗∗*p* < 0.01, ∗∗∗*p* < 0.001, ∗∗∗∗*p* < 0.0001.

We also investigated the impact of model selection and sex-dependent effects on ECG measurements; example traces in Figure 6 illustrate unique ECG morphologies in male and female guinea pigs, across two different age groups. Using the NES-AN approach (Table 8), we observed that the older adult females had significantly faster heart rates compared to male counterparts (Female: 226.3±18.9 bpm; Male: 185.8±2.1 bpm, *p* < 0.05). In lead I, the older males had significantly shorter PR interval (Male: 58.4±1.9 ms, Female: 66.8±6.5 ms, *p* < 0.05), and a longer QT interval (Male: 224.1±10.5 ms; Female: 192.7±15 ms, *p* < 0.05) but not QTc interval (Male: 394.5±20.7 ms; Female: 373±16.3 ms, *p* < 0.1). As shown in Table 8, significant sex-dependent differences in ECG parameters were also observed using other lead configurations.

**TABLE 8:** Sex-dependent effects on ECG parameters using six frontal plane leads in older adult guinea pigs (NES-AN).

Lead	RR(ms)	HR(BPM)	PR(ms)	Pdur(ms)	QRS(ms)	QT(ms)	QTc(ms)	Tpeak-Tend interval (ms)
I(female, n = 4)	266.6±21.1	226.3±18.9	66.8±6.5	24.6±2.4	70.6±14.8	192.7±15	373±16.3	23.6±2.3
I(male, n = 4)	323±3.6	185.8±2.1	58.4±1.9	22.3±1.2	85.7±5.5	224.1±10.5	394.5±20.7	36±4.2
*p* value	**<0.05**	**<0.05**	**<0.05**	0.09	0.07	**<0.05**	0.10	**<0.05**
II(female)			68.7±3.2	29.7±0.1	58.3±2.7	196.5±6.7	384.5±8	22.7±3.5
II(male)			63.3±0.6	32.8±4.2	60.8±3.5	222.5±3.7	391.7±8.2	34±10.9
*p* value			**<0.05**	0.22	0.37	**<0.05**	0.19	0.20
III(female)			60.5±6.3	22.4±3.2	52.4±2.1	191.2±4.9	374.2±11	16.7±7
III(male)			54.8±5.2	22.2±4.7	53.6±3.9	212.7±8.6	374.2±15.7	18.7±4.7
*p* value			0.12	0.47	0.23	**<0.05**	0.30	0.29
aVR(female)			70.8±2.9	32.5±1.9	66.7±1.8	198.4±11.6	387.7±1.4	21.3±0.2
aVR(male)			55.2±5.6	17.5±1.7	41.8±10.9	204.6±11.5	360.1±19.9	18.4±6.4
*p* value			**<0.05**	0.20	**<0.05**	**<0.05**	0.38	**<0.05**
aVL(female)			70.8±2.9	32.5±1.9	66.7±1.8	198.4±11.6	387.7±1.4	21.3±0.2
aVL(male)			55.2±5.6	17.5±1.7	41.8±10.9	204.6±11.5	360.1±19.9	18.4±6.4
*p* value			0.22	0.23	0.44	0.08	0.42	0.41
aVF(female)			65.9±0.9	28.1±0.1	55.2±2.1	195.3±8	382.1±5.4	20.2±5
aVF(male)			61.2±7.7	29.8±2	57.4±6	216.5±6.2	381±11.3	22.8±3.7
*p* value			0.14	0.37	0.27	**<0.05**	0.31	0.25

Values are means ± SD in ms or bpm. *p* value is calculated by homoscedastic Student’s t-test.

*p* values that are statistically significant

In the younger adult females, heart rate was slightly slower than male counterparts (Female: 243.7±10.2 ms; Male: 251.8±21.4, *p* = 0.22) and both the QTc interval (Female: 377.5±18.5 ms; Male: 348.1±14.7 ms, *p* < 0.05), and QT interval were longer (Female: 187.5±11.2 ms; Male: 170.7±13.8 ms, *p* < 0.05) (Table 9). Interestingly, Tpeak-Tend trended longer in the younger adult females (Female: 24.9±5 ms; Male: 14.9±3.9 ms, *p* < 0.05), but shorter in the older adult females compared with age-matched males (Female: 23.6±2.3 ms; Male: 36±4.2 ms, *p* < 0.05). Using the PES-AN approach, heart rate trended slower in both younger and older adult females, compared with age-matched males, however, this parameter did not reach statistical significance (Table 10). The other ECG parameters did not show sex-specific differences in the age-matched groups, when using the platform ECG system (Table 10).

**TABLE 9:** Sex-dependent effects on ECG parameters using six frontal plane leads in younger adult guinea pigs (NES-AN).

Leads	RR(ms)	HR(BPM)	PR(ms)	Pdur(ms)	QRS(ms)	QT(ms)	QTc (ms)	Tpeak-Tend interval (ms)
I(male, n = 6)	240.1±21.2	251.8±21.4	56.8±7.6	23±2.2	67.5±9.1	170.7±13.8	348.1±14.7	14.9±3.9
I(female, n = 7)	246.6±10.2	243.7±10.2	55.5±6.8	22.4±3.4	60±14.3	187.5±11.2	377.5±18.5	24.9±5
*p* value	0.26	0.22	0.39	0.38	0.16	**<0.05**	**<0.05**	**<0.05**
II(male)			54.7±10.4	25.2±2.3	55.7±4.5	184.2±19.1	375.4±26.7	20.6±4.9
II(Female)			57±8.4	28.1±2.6	57.4±5.3	195.6±5.7	393.9±7.8	24±4.5
*p* value			0.34	**<0.05**	0.30	0.10	0.07	0.12
III(male)			45.1±5.5	18.2±1.8	51.7±6.1	182.2±16.9	371.4±19.5	18.8±2.7
III(Female)			42.5±5	18.8±4.2	54.5±3.8	192.6±12.7	387.8±23.4	17.3±3.4
*p* value			0.21	0.39	0.19	0.14	0.12	0.22
aVR(male)			58.2±5.8	23.5±2.2	59.6±6.8	170.2±20.8	346.5±28.7	22.2±6.3
aVR(Female)			52.2±9	26.9±8.7	62.4±4.3	189.9±13.7	382.2±22.1	20.8±4.4
*p* value			0.11	0.20	0.21	**<0.05**	**<0.05**	0.34
aVL(male)			55.2±6.6	17.7±1.1	38±9.1	174.9±25.2	355.8±37.8	14.3±4
aVL(Female)			54.1±6.2	17.3±3.2	41.6±11.9	180.3±13.4	362.8±21.4	17±7.1
*p* value			0.39	0.38	0.29	0.33	0.35	0.23
aVF(male)			52.7±5.3	22.6±2.4	51.9±3.7	182.5±18.6	371.8±22.8	20.8±3.1
aVF(Female)			54.2±5.3	22.9±2.6	51.5±10.5	187.4±9.2	377.3±15.1	21.3±6.5
*p* value			0.32	0.44	0.47	0.29	0.32	0.44

Values are means ± SD in ms or bpm. *p* value is calculated by homoscedastic Student’s t-test.

*p* values that are statistically significant

**TABLE 10:** Sex-dependent effects in ECG parameters using a platform ECG system.

Method/Age	Sex	RR (ms)	HR(BPM)	PR(ms)	Pdur(ms)	QRS(ms)	QT(ms)	QTc(ms)
PES-AN (young)	Male(*n* = 8)	217.2±17.7	278±21.2	48.7±5.4	36.6±6	45.9±7.9	156.8±15.6	336.1±22.6
	Female(*n* = 8)	234.3±24.2	260.1±28	49.3±4.2	34.9±3.6	41.6±7	162.5±13.3	336±12.7
	*p* value	0.07	0.09	0.41	0.26	0.14	0.23	0.49
PES-AN (old)	Male(*n* = 5)	270.7±26.4	224±22.2	50.3±10.7	37±7.5	71.8±14.6	188±17.4	361.5±26.5
	Female(*n* = 9)	292.4±43	209.9±29.1	57.7±3.7	38.7±4.5	66.7±11.3	193.1±26.9	357±28.2
	*p* value	0.11	0.11	0.13	0.44	0.48	0.19	0.34
PES-AW (young)	Male(*n* = 4)	222.3±24	273.9±29.6	60.2±3.5	38±4	51.1±4.4	140±6.4	297.7±4.3
	Female(*n* = 3)	210.4±18.3	288.3±25.6	58±4.4	39.3±5.4	41±8.1	127.6±15.7	277.4±22.8
	*p* value	0.27	0.29	0.28	0.39	0.08	0.16	0.12

Values are means ± SD in ms or bpm. *p* value is calculated by homoscedastic Student’s t-test.

## Discussion

In this study, we investigated whether ECG measurements are modulated by methodological or demographic variability. *In vivo* ECGs were collected from guinea pigs (n = 30) using different recording techniques while animals were awake or anesthetized. Needle ECG recordings (lead I and lead II) were used to construct frontal plane leads (lead III, aVL, aVR, aVF), and we tested whether lead configuration contributed to variability in ECG metric measurements. To verify the presence of demographic heterogeneity on ECG measurements, we classified animals into age- and sex-specific groups.

### Methodological Heterogeneity Alters ECG Measurements

Our data suggest that different ECG recording techniques, lead configuration, and the presence or absence of anesthetics can significantly affect ECG metric measurements. Several ECG metrics obtained using PES-AW, PES-AN, and NES-AN showed significant differences according to methodological approach (Figure 2; Table 1, Table 2). In an age and sex-matched guinea pig population, it is evident that isoflurane anesthesia slows heart rate compared to conscious animals (Figure 2). Further, repolarization time (QT interval and QTc) was also longer in NES-AN recordings, as compared to either PES-AN or PES-AW (Table 1). These results indicate that ECG measurements can vary significantly depending on the recording technique.

The majority of our ECG measurements were consistent with previously reported ECG values in both conscious (Hess et al., 2007) and anesthetized (Brouillette et al., 2007; Kijtawornrat et al., 2011; Orvos et al., 2020) guinea pigs. Although, we did note that our QRS duration measurements matched reported values in some studies from conscious (Botelho et al., 2016) and anesthetized (Andršová et al., 2020) guinea pigs, but were longer than reported values for other studies (Brouillette et al., 2007)^,^ (Hess et al., 2007)^,^ (Hancock et al., 2009). The difference in reported values may be attributed to the definition of the QRS duration; in our study example ECG traces showed a prominent S wave in the PES-AN and NES-AN measurements that yielded a longer QRS duration (Figure 2).

In addition to ECG recording techniques, we also found that measured parameters varied significantly depending on the presence or absence of anesthesia (Table 3). This finding is in agreement with an earlier study that demonstrated a wider RR and QT interval in anesthetized versus conscious guinea pigs (Hamlin et al., 2003). The effect of anesthesia on ECG parameters has been documented in other experimental models, including mice (Warhol et al., 2021), as well as the impact of alternative anesthetic agents (Ho et al., 2011). Similarly, clinical studies (Kazanci et al., 2009) have also reported that anesthesia impacts ECG measurement values. Since anesthetics are a strong confounding factor in ECG values, care should be taken to maintain a consistent level of anesthesia between animals in order to reduce additional heterogeneity in ECG signal measurements (Yang et al., 2014).

Electrophysiology measurements were also shown to vary according to the ECG lead configuration (Supplementary Table S1-S2). Accordingly, such variability in the same ECG parameter value, due to lead configuration, has been reported in clinical studies (; Baumert et al., 2016). Electrical impulse propagation within the heart is a spatiotemporal variable and the signal strength realized from a specific lead can vary depending on the lead location and wavefront location at a given time, causing considerable inter-lead variability in ECG parameter values. Within experimental studies, care should be taken to accurately maintain the lead configuration between animals—and also to consider lead configuration when comparing reported ECG values across experimental studies.

### Demographic Heterogeneity Impacts EGG Measurements

One of the objectives of this study was to test if demographic heterogeneity, due to animal model selection, results in diverse ECG parameter values. In the presented study, age- and sex-specific differences in ECG measurements were documented.

First, our study suggests that ECG metric measurements are strongly modified by age. The majority of the ECG parameters measured in this study varied significantly between younger and older adult guinea pigs. Notably, these ECG differences were preserved across multiple methodologies (Figure 3, Figure 5, Figure 6 and Table 4, Table 5, Table 6). Even though VCG parameters did not show a significant difference between the two age groups (Table 7), we did observe a left-upward shift of the QRS loops and vectors in the older versus younger adults (Figure 5D). This finding is consistent with previous studies (Brisinda et al., 2007; Shiotani et al., 2008) that report longer a RR and PR interval, and a slight wider QRS complex in older versus younger animals. Similar observations have been reported in other animal models, including older rats, which present with a slower heart rate and longer ECG duration parameters (RR, PR, QRS and QT) (Rossi et al., 2014). Finally, the results of our *in vivo* ECG measurements are consistent with *in vitro* studies using isolated cardiomyocytes, which display longer APD50 and APD80 values in adult versus neonatal guinea pig myocytes (Kato et al., 1996). In our study, we found that guinea pig age was a significant modulator of ECG measurements (independent of sex). To improve the reproducibility of electrophysiology studies, care should be taken to clearly define the age of experimental animal models.

We also found sex-specific effects on ECG parameter values, albeit in an age-dependent fashion. Sex-specific ECG effects were observed using needle ECG recordings—but not the platform ECG recording technique, which further supports our argument that methodological variability impacts the ECG metric measurements. Sex-specific effects on ECG measurements were primarily reflected in heart rate, which was faster in older adult females compared to males. Inversely, younger adult females had a slightly slower heart rate than age-matched males (Figure 6). As shown in Figure 6C, in the older adult group (lead I), the QT interval in males was significantly longer than the females, while in the young group the QT interval was shorter in the males. We noted a similar trend in the Tpeak-Tend measurement, which was longer in the younger adult females, but shorter in the older females compared to male counterparts. Another interesting aspect of this comparison was that male guinea pigs had age-dependent changes in multiple ECG parameters (heart rate, QT, QTc and Tpeak-Tend), but only slight changes were observed in female guinea pigs of differing ages.

Collectively, the aforementioned findings suggest sex modifies ECG parameters in guinea pigs—but, that these effects are likely age dependent. As such, significant sex-specific differences may be present in one age group, but abolished in another age group. In our study, age was found to more strongly influence ECG parameters in males, as compared to females. Of interest, Brouillette et al. previously reported that sex-specific effects did not impact the ECGs recorded from guinea pigs (Brouillette et al., 2007). Although slight differences in the anesthetic agent could contribute to this finding—as this study used pentobarbital, which can exert a stronger cardiodepressive effect (Ho et al., 2011) that could mask sex-specific differences in the ECG signal. Although Brouillette, et al. did detect a trend toward longer QT, QTc, and faster heart rates in younger females—which is consistent with our findings in our younger adult group (Brouillette et al., 2007). A longitudinal study by Brisinda, et al. found that older female guinea pigs (14 months) had longer PR intervals, shorter QTc interval, and shorter Tpeak-Tend compared with age-matched males–consistent with findings in our older adult group (Brisinda et al., 2007). These findings support our conclusion that sex-specific differences in the ECG signals may be strongly influenced by the age of the animal groups.

### Significance of the Current Study

Guinea pig ECG parameter values reported here are in agreement with previous findings, in both conscious (Hamlin et al., 2003; Hess et al., 2007) and anesthetized (Brouillette et al., 2007; Kijtawornrat et al., 2011) animals—which demonstrate the robustness of our approach. Slight differences in our reported ECG values compared to other studies could be attributed to diverse ECG recording methodologies, including implantable telemetry, needle, and platform-based systems. Results may also differ according to the presence or absence of anesthetics, depth of anesthesia, and ECG lead configuration. We also demonstrated that animal model selection can induce heterogeneity in ECG metrics, with both the age and sex of animals acting as confounding factors.

The implication of our study is important, as ECGs are widely used to assess *in vivo* electrophysiology in animal models. ECG-derived biomarkers are commonly employed to evaluate the safety and efficacy of drugs, and to investigate cardiovascular disease using experimental models (Ho et al., 2011). Although the usefulness of ECG biomarkers is significantly reduced if signal heterogeneity is not quantified and addressed. For example, QT prolongation is a well-known biomarker for pre-clinical antiarrhythmic drug assessment (Shiotani et al., 2007) or to evaluate pro-arrhythmic conditions (e.g., long QT syndrome) (Salama and Bett, 2014). In this study, we demonstrated that QT interval measurements can vary widely depending on the ECG recording technique, use of anesthesia, lead configuration, or demographic characteristics of the animal model. This highlights the need to better standardize ECG biomarkers in animal models (Quinn et al., 2011; Baumert et al., 2016), and our study emphasizes the need for detailed reporting of ECG methodologies and animal characteristics to allow for comparisons between studies.

Finally, our study may have important implications for the development of ECG AI algorithms, which have been developed for the clinical assessment of ECG or cardiovascular risk stratification (Haq et al., 2020; Siontis et al., 2021). The efficiency of AI algorithms depends heavily on the robustness of the corresponding training dataset; this can pose challenges for the development of ECG tools, if only a common database is used then it could lead to erroneous ECG analysis and interpretation. For example, using AI algorithms to evaluate a lead II ECG trace may prove unsuccessful if the training dataset was built using predominately lead I recordings. Care should be taken to utilize diverse ECG databases with adequate information on the heterogeneity of key variables (e.g., recording method, lead configuration, age, sex).

### Limitations

In the presented study, we used different ECG recording techniques (needle and platform-based), albeit additional technical approaches are available that were not explored in this study (e.g., telemetry). We also did not assess multiple depths of anesthesia, different anesthetic agents, or the effect of shortened or prolonged anesthetic exposure time on ECG metrics. In our study, we used a similar level (3–4% isoflurane) and length of anesthesia-induction (10 min) for each animal, but we only considered anesthesia as a binary variable (presence or absence). Finally, we only used limb lead recordings to obtain ECG and VCG measurements, and our frontal plane VCG metrics did not achieve statistical significance in the one specific plane investigated. More robust measurements of 3D electrical impulse propagation patterns could be achieved by incorporating precordial lead recordings (lead V1-V6) and constructing a sagittal plan projection.

## Data Availability

The raw data supporting the conclusion of this article will be made available by the authors, without undue reservation.

## Abbreviations

AI, artificial intelligence; ECG, electrocardiogram; NES-AN, needle ECG system, anesthetized animals; PES-AN, platform ECG system, anesthetized animals; PES-AW, platform ECG system, awake animals; VCG, vectorcardiogram.
