# Supplementary material for: Demographic and Methodological Heterogeneity in Electrocardiogram Signals From Guinea Pigs
Source: Front Physiol. 2022 Jun 2;13:925042. doi: 10.3389/fphys.2022.925042 (PMC9202081; doi:10.3389/fphys.2022.925042)
Supplement: Supplementary file 1 [file Table1.docx]

**Supplemental Table S1, A**: Effect of inter-lead variability on ECG parameters in younger adult guinea pigs (n=13, NES-AN)

| **Leads** | **RR(ms)** | **HR(BPM)** | **PR(ms)** | **Pdur(ms)** | **QRS(ms)** | **QT(ms)** | **QTc (ms)** | **Tpeak Tend Interval (ms)** |
| --- | --- | --- | --- | --- | --- | --- | --- | --- |
| I | 243.6±16.6 | 247.4±16.9 | 56.1±7.2 | 22.7±2.9 | 63.5±12.8 | 179.7±15 | 363.9±22.3 | 20.3±6.7 |
| II |  |  | 55.9±9.5 | 26.7±2.9 | 56.6±5 | 190.3±14.8 | 385.4±21.1 | 22.4±5 |
| III |  |  | 43.7±5.4 | 18.5±3.3 | 53.2±5.2 | 187.8±15.7 | 380.3±23.2 | 18±3.1 |
| aVR |  |  | 55±8.2 | 25.3±6.8 | 61.1±5.8 | 180.8±19.9 | 365.7±30.9 | 21.5±5.4 |
| aVL |  |  | 54.6±6.4 | 17.5±2.5 | 39.9±10.9 | 177.8±19.9 | 359.6±30.3 | 15.8±6 |
| aVF |  |  | 53.5±5.3 | 22.8±2.6 | 51.7±8.1 | 185.1±14.5 | 374.8±19.2 | 21.1±5.2 |

Values are means ± SD in ms or bpm.

**Supplemental Table S1, B**: p values of inter-lead comparisons in ECG parameters in younger adult guinea pigs (n=13, NES-AN)

| **Groups** | **RR(ms)** | **HR(BPM)** | **PR(ms)** | **Pdur(ms)** | **QRS(ms)** | **QT(ms)** | **QTc (ms)** | **Tpeak Tend Interval (ms)** |
| --- | --- | --- | --- | --- | --- | --- | --- | --- |
| I-II | ns | ns | ns | **<0.05** | **<0.05** | **<0.05** | **<0.05** | ns |
| i-iii | ns | ns | **<0.0001** | **<0.05** | **<0.05** | ns | **<0.05** | ns |
| I-aVR | ns | ns | ns | ns | ns | ns | ns | ns |
| i-aVL | ns | ns | ns | **<0.0001** | **<0.0001** | ns | ns | **<0.05** |
| i-aVF | ns | ns | ns | ns | **<0.05** | ns | ns | ns |
| ii-iii | ns | ns | **<0.001** | **<0.0001** | ns | ns | ns | **<0.05** |
| ii-aVR | ns | ns | ns | ns | **<0.05** | ns | **<0.05** | ns |
| ii-aVL | ns | ns | ns | **<0.0001** | **<0.0001** | **<0.05** | **<0.05** | **<0.05** |
| ii-aVF | ns | ns | ns | ns | ns | ns | ns | ns |
| iii-aVR | ns | ns | **<0.001** | **<0.001** | **<0.001** | ns | ns | **<0.05** |
| iii-aVL | ns | ns | **<0.0001** | ns | **<0.001** | ns | **<0.05** | ns |
| iii-aVF | ns | ns | **<0.001** | **<0.0001** | ns | ns | ns | ns |
| aVR-aVL | ns | ns | ns | **<0.001** | **<0.0001** | ns | ns | **<0.05** |
| aVR-aVF | ns | ns | ns | ns | **<0.05** | ns | ns | ns |
| aVL-aVF | ns | ns | ns | **<0.0001** | **<0.05** | ns | ns | **<0.05** |

p value is calculated by homoscedastic Student’s t-test.
