# Supplementary material for: Demographic and Methodological Heterogeneity in Electrocardiogram Signals From Guinea Pigs
Source: Front Physiol. 2022 Jun 2;13:925042. doi: 10.3389/fphys.2022.925042 (PMC9202081; doi:10.3389/fphys.2022.925042)
Supplement: Supplementary file 2 [file Table2.docx]

**Supplemental Table S2, A**: Effect of inter-lead variability on ECG parameters in older adult guinea pigs (n=9, NES-AN)

| **Leads** | **RR(ms)** | **HR(BPM)** | **PR(ms)** | **Pdur(ms)** | **QRS(ms)** | **QT(ms)** | **QTc (ms)** | **Tpeak Tend Interval (ms)** |
| --- | --- | --- | --- | --- | --- | --- | --- | --- |
| I | 304.3±31.9 | 200.7±24.6 | 62.9±6.6 | 24.1±2.3 | 77.8±12.4 | 211.5±19 | 383.7±19.6 | 33.2±6.4 |
| II |  |  | 65.2±3.1 | 31.8±3.8 | 59.1±3.3 | 214.3±13.5 | 390.1±9 | 28.5±10 |
| III |  |  | 55.2±6.7 | 22.6±4.3 | 54.3±3.7 | 208.7±14.6 | 379.9±14.9 | 19±5.8 |
| aVR |  |  | 66.5±3.7 | 30.3±3.9 | 63.9±2.5 | 214.3±14 | 390±10.3 | 25.9±4.3 |
| aVL |  |  | 54.9±6.7 | 18.9±2.6 | 46.5±10.1 | 204.7±14 | 372.7±16.5 | 18.3±5.4 |
| aVF |  |  | 60.5±7.3 | 28.5±1.6 | 57.5±5.3 | 211.9±13.6 | 385.6±10 | 22.3±4.4 |

Values are means ± SD in ms or bpm.

**Supplemental Table S2, B**. p values of inter-lead comparisons in ECG parameters in older adult guinea pigs (n=9, NES-AN)

| **Groups** | **RR(ms)** | **HR(BPM)** | **PR(ms)** | **Pdur(ms)** | **QRS(ms)** | **QT(ms)** | **QTc (ms)** | **Tpeak Tend Interval (ms)** |
| --- | --- | --- | --- | --- | --- | --- | --- | --- |
| I-II | ns | ns | ns | **<0.05** | **<0.05** | ns | ns | ns |
| i-iii | ns | ns | **<0.05** | ns | **<0.001** | ns | ns | **<0.05** |
| I-aVR | ns | ns | ns | **<0.05** | **<0.05** | ns | ns | **ns** |
| i-aVL | ns | ns | **<0.05** | **<0.05** | **<0.001** | ns | ns | **<0.05** |
| i-aVF | ns | ns | ns | **<0.05** | **<0.05** | ns | ns | **<0.05** |
| ii-iii | ns | ns | **<0.05** | **<0.05** | **<0.05** | ns | ns | **<0.05** |
| ii-aVR | ns | ns | ns | ns | **<0.05** | ns | ns | ns |
| ii-aVL | ns | ns | **<0.05** | **<0.0001** | **<0.05** | ns | **<0.05** | **<0.05** |
| ii-aVF | ns | ns | ns | ns | ns | ns | ns | ns |
| iii-aVR | ns | ns | **<0.05** | **<0.05** | **<0.05** | ns | ns | **<0.05** |
| iii-aVL | ns | ns | ns | ns | ns | ns | ns | ns |
| iii-aVF | ns | ns | ns | **<0.05** | ns | ns | ns | ns |
| aVR-aVL | ns | ns | ns | ns | **<0.05** | ns | ns | ns |
| aVR-aVF | ns | ns | ns | ns | **<0.05** | ns | ns | ns |
| aVL-aVF | ns | ns | ns | **<0.0001** | **<0.05** | ns | ns | ns |

p value is calculated by homoscedastic Student’s t-test.
