# Supplementary material for: Demographic and Methodological Heterogeneity in Electrocardiogram Signals From Guinea Pigs
Source: Front Physiol. 2022 Jun 2;13:925042. doi: 10.3389/fphys.2022.925042 (PMC9202081; doi:10.3389/fphys.2022.925042)

Supplemental Figure S1: Comparison of ECG parameters collected at 3 different time points, using a platform ECG system.

S1

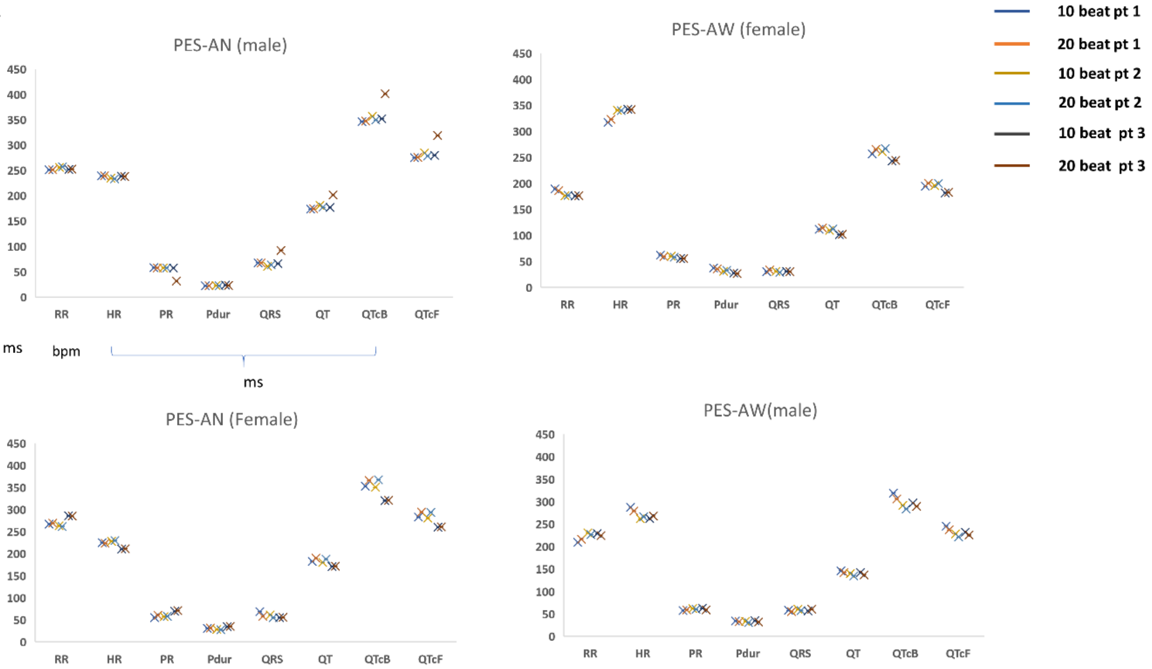

Supplement: Supplementary file 3 [file Image1.pdf]
